# 2D sodium MRI of the human calf using half‐sinc excitation pulses and compressed sensing

**DOI:** 10.1002/mrm.29841

**Published:** 2023-10-05

**Authors:** Rebecca R. Baker, Vivek Muthurangu, Marilena Rega, Javier Montalt‐Tordera, Samuel Rot, Bhavana S. Solanky, Claudia A. M. Gandini Wheeler‐Kingshott, Stephen B. Walsh, Jennifer A. Steeden

**Affiliations:** ^1^ UCL Centre for Translational Cardiovascular Imaging University College London London UK; ^2^ Institute of Nuclear Medicine University College Hospital London UK; ^3^ NMR Research Unit, Queen Square MS Centre, Department of Neuroinflammation, UCL Queen Square Institute of Neurology, Faculty of Brain Sciences University College London London UK; ^4^ Department of Medical Physics and Biomedical Engineering University College London London UK; ^5^ Department of Brain and Behavioral Sciences University of Pavia Pavia Italy; ^6^ Digital Neuroscience Research Unit IRCCS Mondino Foundation Pavia Italy; ^7^ Department of Renal Medicine University College London London UK

**Keywords:** compressed sensing, half‐sinc, ^23^Na MRI, sodium, UTE

## Abstract

**Purpose:**

Sodium MRI can be used to quantify tissue sodium concentration (TSC) in vivo; however, UTE sequences are required to capture the rapidly decaying signal. 2D MRI enables high in‐plane resolution but typically has long TEs. Half‐sinc excitation may enable UTE; however, twice as many readouts are necessary. Scan time can be minimized by reducing the number of signal averages (NSAs), but at a cost to SNR. We propose using compressed sensing (CS) to accelerate 2D half‐sinc acquisitions while maintaining SNR and TSC.

**Methods:**

Ex vivo and in vivo TSC were compared between 2D spiral sequences with full‐sinc (TE = 0.73 ms, scan time ≈ 5 min) and half‐sinc excitation (TE = 0.23 ms, scan time ≈ 10 min), with 150 NSAs. Ex vivo, these were compared to a reference 3D sequence (TE = 0.22 ms, scan time ≈ 24 min). To investigate shortening 2D scan times, half‐sinc data was retrospectively reconstructed with fewer NSAs, comparing a nonuniform fast Fourier transform to CS. Resultant TSC and image quality were compared to reference 150 NSAs nonuniform fast Fourier transform images.

**Results:**

TSC was significantly higher from half‐sinc than from full‐sinc acquisitions, ex vivo and in vivo. Ex vivo, half‐sinc data more closely matched the reference 3D sequence, indicating improved accuracy. In silico modeling confirmed this was due to shorter TEs minimizing bias caused by relaxation differences between phantoms and tissue. CS was successfully applied to in vivo, half‐sinc data, maintaining TSC and image quality (estimated SNR, edge sharpness, and qualitative metrics) with ≥50 NSAs.

**Conclusion:**

2D sodium MRI with half‐sinc excitation and CS was validated, enabling TSC quantification with 2.25 × 2.25 mm^2^ resolution and scan times of ≤5 mins.

## 1 INTRODUCTION

Tissue sodium concentration (TSC) is a potential quantitative biomarker of diseases that affect sodium homeostasis (e.g., chronic kidney disease^1^ and hypertension^2^). Sodium (^23^Na) MRI is a noninvasive method of quantifying TSC and can detect abnormal TSC in leg skin or muscle of patients with multiple sclerosis,^3^ salt‐losing tubulopathies,^4^ and muscular dystrophies.^5^ However, ^23^Na MRI is limited by two main challenges: (i) poor SNR, due to low in vivo sodium concentrations and low intrinsic sensitivity; and (ii) rapid biexponential signal decay, which necessitates UTE sequences.

Typically, 3D sequences are used for ^23^Na MRI because the TE can be minimized using nonselective hard pulses.^6^, ^7^, ^8^, ^9^ However, in some applications (e.g., skin^10^ or heart^11^), 2D imaging may be preferable to enable higher in‐plane resolution, shorter scan times, or quicker image reconstruction. The main disadvantage of 2D imaging is longer TEs, due to the requirement for slice‐select and slice‐select‐rewinder gradients.

**FIGURE 1:**
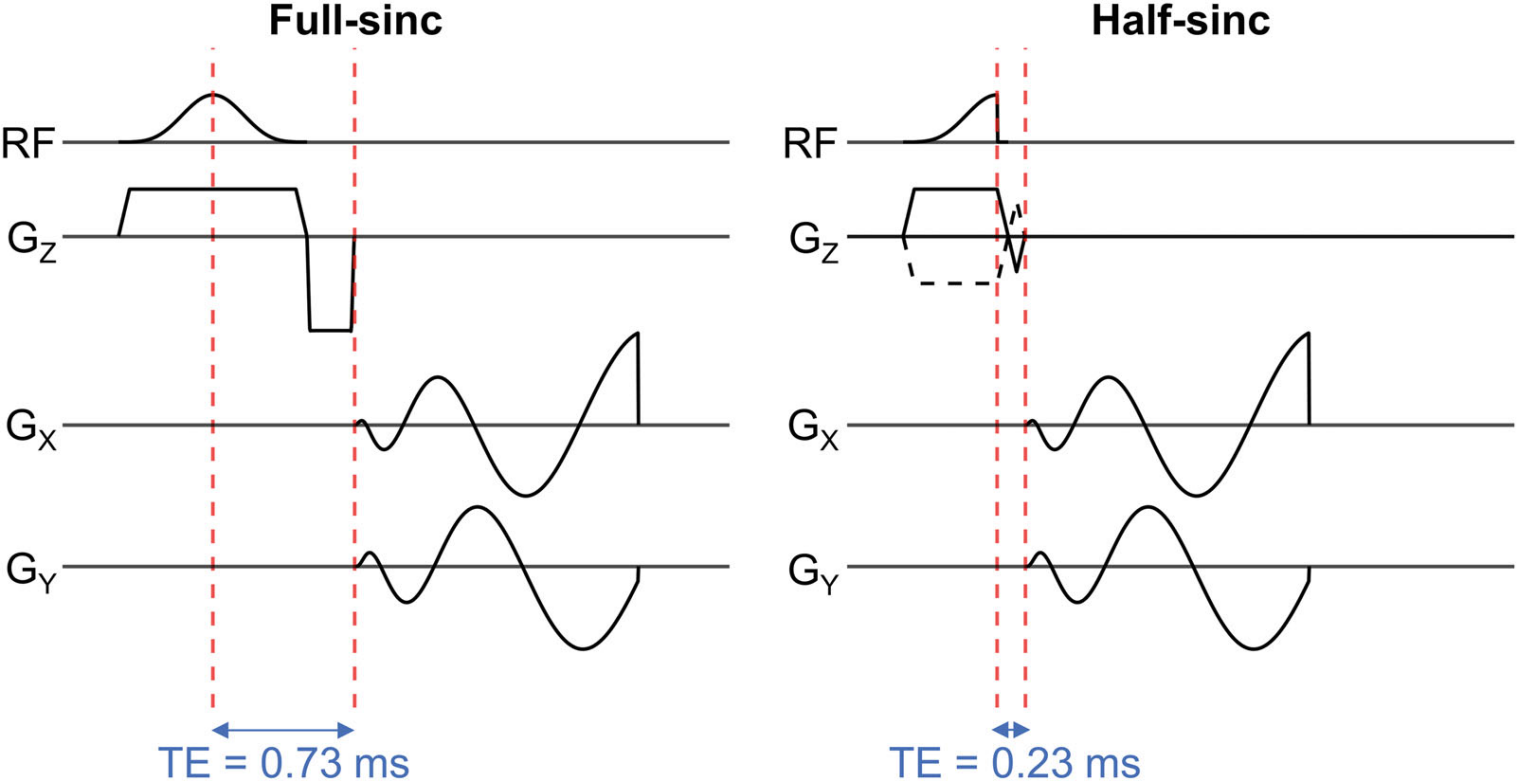
Pulse sequence diagrams for 2D sodium imaging with a center‐out spiral k‐space trajectory and a single‐lobe full‐sinc excitation pulse (left) or a half‐sinc excitation pulse (right). The TE is reduced from 0.73 ms with the full‐sinc pulse, and to 0.23 ms with the half‐sinc pulse; however, with the half‐sinc pulse it is necessary to acquire data across two acquisitions, with slice‐select gradients of opposite polarity. These two acquisitions are summed to give the same slice profile as the full‐sinc. G_Z_/G_X_/G_Y_, magnetic field gradients applied along the Z, X, and Y axes, respectively, where Z is the slice‐select axis and X and Y lie in the plane of the imaging slice; RF, radiofrequency excitation pulse.

Half‐pulse RF excitations^12^ can potentially overcome this but are rarely used in sodium imaging.^13^, ^14^, ^15^ In this approach, two acquisitions are performed with the same readout gradients but half‐sinc excitations with slice‐select gradients of opposite polarity (Figure 1). The two acquisitions are subsequently added to give the same slice profile as the full‐sinc excitation. Although half‐sinc excitation results in a significant reduction in TE, overall scan time is doubled. This can be compensated by reducing the number of signal averages (NSAs), at a cost to SNR. Recently, compressed sensing (CS) has been shown to improve SNR in sodium imaging,^16^, ^17^, ^18^ but only a small number of studies have applied it in the leg.^19^, ^20^

The focus of this study was to develop a high‐resolution 2D UTE–^23^Na MRI sequence with CS reconstruction for rapid and accurate TSC quantification in skeletal muscle and skin. Specifically, the aims were: (1) to develop a 2D UTE sequence with half‐sinc excitation for sodium imaging, (2) to optimize a CS reconstruction for accurate sodium quantification with reduced scan time, and (3) to validate the developed techniques ex vivo and in vivo by assessing TSC accuracy and image quality.

## 2 METHODS

### 2.1 Sequence design

We developed a 2D uniform‐density center‐out spiral sodium sequence using the spiral algorithm by Pipe and Zwart.^21^ Sequence parameters were: FOV = 180 × 180 mm^2^, pixel size = 2.25 × 2.25 mm^2^, slice thickness = 30 mm, TR = 100 ms, spiral readout duration = 7.6 ms, 20 regularly spaced spirals for complete k‐space filling, and 150 NSAs.

### 2.2 Full‐sinc and half‐sinc 2D excitation pulses

A single‐lobe, full‐sinc RF pulse was designed (flip angle = 90°, pulse duration = 600 μs), resulting in TE = 0.73 ms. Splitting this pulse for half‐sinc excitation (pulse duration = 300 μs) resulted in TE = 0.23 ms (Figure 1). Total scan time was 5:07 min:s and 10:15 min:s for 2D full‐sinc and half‐sinc acquisitions, respectively.

### 2.3 Imaging experiments

All imaging was performed on a multinuclear 3 T MRI scanner (Biograph mMR, Siemens Healthineers, Erlangen, Germany). A transmit/receive ^23^Na birdcage coil (Stark Contrast, Erlangen, Germany) was positioned around the tissue. Five calibration phantoms with nominal sodium concentrations of 10, 25, 50, 75, and 100 mM were placed on top of the tissue within the coil and image FOV. Phantoms were fixed in parallel foam sleeves, and care was taken to ensure they remained orthogonal to the imaging plane. Manual B_0_‐field shimming and flip‐angle calibration were performed separately for 2D and 3D acquisitions within a shim volume covering the tissue and calibration phantoms in the imaging volume.

#### 2.3.1 Ex vivo imaging

In three ex vivo porcine samples, with one sample repeated twice, 2D sodium acquisitions with full‐sinc and half‐sinc excitation were compared to a reference 3D acquisition‐weighted stack‐of‐spirals sequence^22^ with a nonselective excitation pulse (duration = 250 μs). The spiral trajectories, in‐plane FOV, in‐plane resolution, and TR were the same as the 2D sequences. Other 3D sequence parameters were slice thickness = 15 mm, slice partitions = 6, TE = 0.22 ms, NSAs = 120, total scan time = 24 min. Muscle TSC was quantified by averaging over two consecutive slices for sufficient SNR and to match the slice thickness of the 2D experiments.

#### 2.3.2 In vivo imaging

Ten healthy volunteers were enrolled in the study (5 male, 5 female; mean age 30 ± 6 years) and provided written consent. This study was approved by the local research ethics committee (Ref. 15/0041). Sodium imaging was performed on the right calf using 2D acquisitions with full‐sinc and half‐sinc excitation and the same setup as the ex vivo experiment. TSC was quantified in the muscle and skin.

### 2.4 Image reconstruction

Conventional reconstruction for 2D and 3D acquisitions consisted of nonuniform fast Fourier transform (NUFFT) with density compensation.^23^, ^24^

In vivo 2D half‐sinc data with retrospectively reduced NSAs was also reconstructed using CS by solving the following minimization problem^25^:

(1)

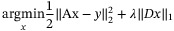


Where x is the desired image, A is the encoding matrix (which includes Fourier transform and sampling matrix), y is the measured data, D is a sparsifying transform, and λ is a regularization weighting parameter. The first term enforces data consistency, whereas the second term promotes sparsity in a transform domain.

Optimal regularization was investigated by reconstructing reduced NSAs data (15, 30, 40, 50, 60, 75) at different values of λ (0.0, 0.1, 0.3, 0.5, 0.7, 1.0, 1.3, 1.6, 3.2, 5.0), using a total variation transform. Optimal λ for a given NSAs was chosen by comparing the SD of pixels in a muscle region of interest (ROI) in TSC maps from CS (SD_CS_) with reference 150 NSAs NUFFT maps (SD_Ref_), using SD_CS_/SD_Ref_. Specifically, for a given NSAs, the optimal λ was taken where SD_CS_/SD_Ref_ was closest to 1.0 (i.e., the most similar pixel distribution to the reference map), minimizing noise (observed where SD_CS_/SD_Ref_ >1) and excess smoothing (observed where SD_CS_/SD_Ref_ <1).

All CS reconstructions were developed in‐house using Python (version 3.8.10) and TensorFlow MRI (version 0.21.0).^24^

### 2.5 TSC quantification

ROIs were drawn in the center of each reference phantom (to avoid partial volume effects), and a linear calibration was performed (Supporting Information S1). TSC maps were computed in MatLab (R2022a, MathWorks, Inc., Natick, MA), and results are reported as apparent TSC (aTSC),^26^ quantified as the mean value from ROIs drawn using ITK‐SNAP (version 3.8.0)^27^ (Supporting Information S2).

Due to the rapid decay of the sodium signal, TE can have a significant effect on aTSC accuracy. The sodium signal over time, *t*, can be described by:^28^, ^29^

(2) signal≈1−exp−TRT1×cSexp−tT2S+cLexp−tT2L

Where $c_{S}:c_{L}$ are the relative proportions of the short T_2_ ($T_{2S}$) and long T_2_ ($T_{2L}$) components. This equation can be used to model the effect of calibration phantom and tissue relaxation on signal amplitude, as well as to perform relaxation correction of the acquired signal (where *t* = TE). In silico modeling used normalized phantom and muscle signals, and the ratio of the two was used as a proxy for aTSC (with a ratio of 1 being most accurate). Because different tissue types and tissues affected by disease may not exhibit the same relaxation properties, it is to be expected that properties of calibration phantoms and tissues may not exactly match. However, relaxation correction can be performed if these parameters are known. Properties of the phantoms used for these experiments have been reported by Rot et al.^30^: $c_{S}:c_{L}$ = 0.7:0.3, $T_{2S}$ = 4–6 ms, $T_{2L}$ = 17–24 ms, and $T_{1}$ = 27–32 ms. However, measurement of individual tissue relaxation parameters was not feasible; hence, published values were used. Specifically, for demonstration, correction was performed on ex vivo data using literature values for muscle^31^: $c_{S}:c_{L}$ = 0.6:0.4, $T_{2S}$ = 1.5–2.5 ms, $T_{2L}$ = 15–30 ms, and $T_{1}$ = 12–25 ms^30^; results are reported with and without relaxation correction and compared to modeled signals. Phantom and muscle relaxation properties are described further in Supporting Information S3.

### 2.6 Image quality assessment

In vivo quantitative and qualitative metrics were measured to assess the effect of reduced NSAs and CS on the quality of 2D half‐sinc images. Quantitative image quality was assessed using estimated SNR and edge sharpness (ES) measures. Calculation of SNR in images reconstructed using CS is nontrivial due to the uneven distribution of noise. Therefore, SNR was estimated in the images using mean signal intensity in an ROI in tissues of interest and SD of noise from a background ROI (Supporting Information S2).^32^

ES was calculated by measuring the maximum gradient of pixel intensities across the 100 mM phantom border on aTSC maps (chosen as the phantom with the highest signal, limiting artificially high edges caused by noise). Pixel intensities were filtered using a Savitzky–Golay filter to remove the effect of noise (window width = 15 pixels, second‐order polynomial) before differentiation. ES was taken as the maximum gradient of the filtered pixel intensities (Supporting Information S2) and was calculated in four positions around the phantom, with the average value used for comparison. Estimated SNR and ES were computed in MatLab (MathWorks).

Qualitative image quality was assessed in two categories using a 5‐point Likert scale: ability to identify separate structures of the leg, such as skin and muscle (1 = very difficult, 2 = difficult, 3 = okay, 4 = easy, 5 = very easy), and perceptual noise (1 = very high, 2 = high, 3 = moderate, 4 = low, 5 = very low). Scoring was performed independently by two observers (v.m. and m.r.), and images were fully anonymized and presented in a random order.

### 2.7 Statistical analysis

In vivo full‐sinc and half‐sinc aTSC were compared using a paired t‐test. aTSC and image quality metrics from half‐sinc acquisitions with fewer NSAs were compared between NUFFT/CS reconstructed data and reference data using analysis of variance, followed by post hoc comparisons using Tukey's test. All data are presented as mean ± SD. Results were considered statistically significant for *p* < 0.05. All statistical analysis was performed using RStudio (Version 2022.7.2.576, RStudio, PBC, Boston, MA, USA).

## 3 RESULTS

^23^Na MRI data was successfully collected with full‐sinc and half‐sinc excitation, both ex vivo (in addition to the 3D sequence, *N* = 4) and in vivo (*N* = 10).

### 3.1 Comparison of full‐sinc and half‐sinc excitation

#### 3.1.1 Ex vivo imaging

**FIGURE 2:**
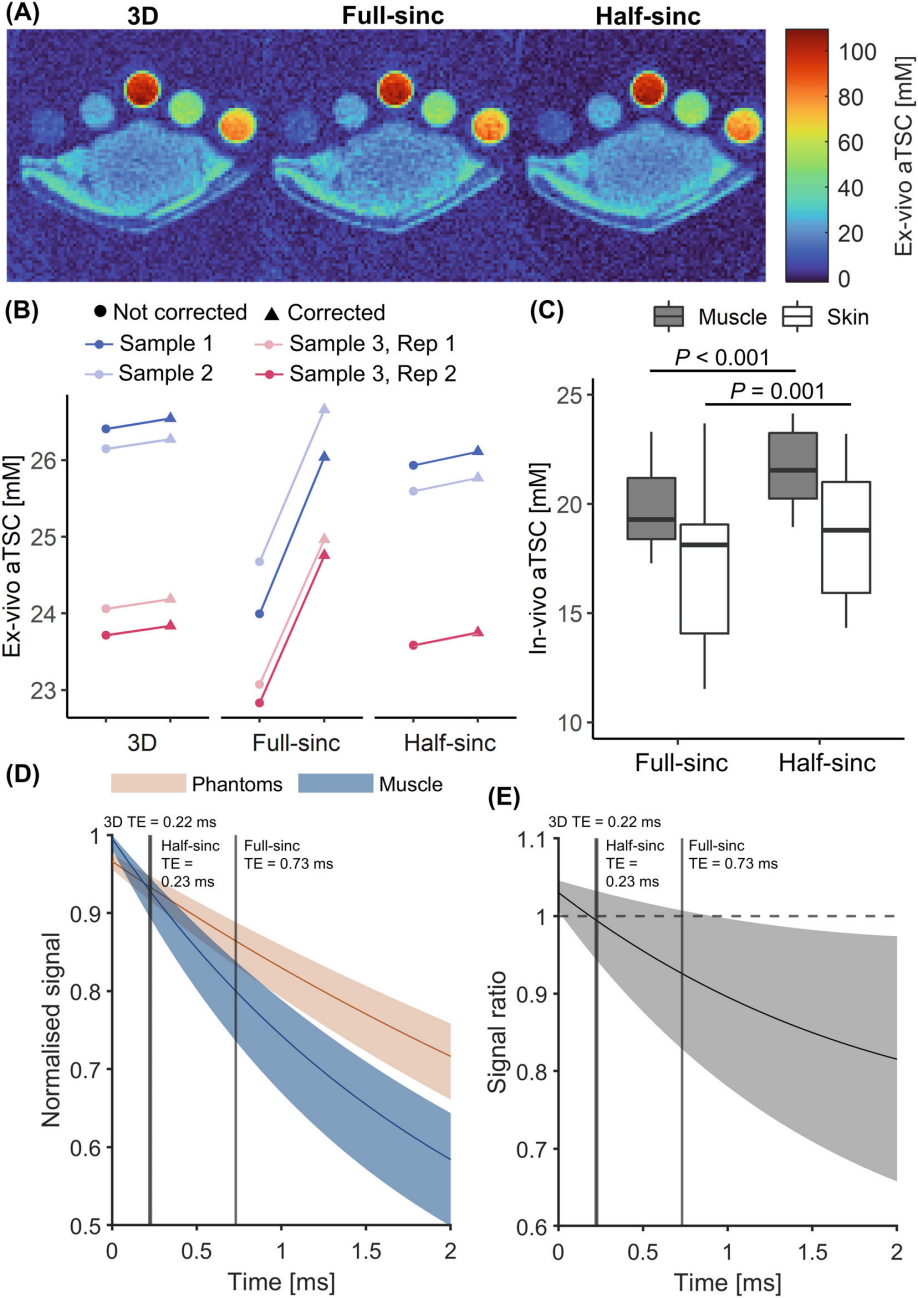
(A) Example ex vivo aTSC maps quantified from a 3D acquisition‐weighted stack‐of‐spirals sequence and 2D acquisitions with full‐sinc and half‐sinc excitation. (B) Ex vivo muscle aTSC quantification. Values are presented without relaxation correction (circles, left) and with relaxation correction (triangles, right) for three samples, plus one repeated sample. (C) Boxplots showing in vivo aTSC in the muscle and skin, quantified from 2D acquisitions with full‐sinc and half‐sinc excitation (*N* = 10, NSAs = 150). *p*‐value calculated between full‐sinc and half‐sinc data using a paired *t*‐test. (D) In silico modeling of signal relaxation in sodium phantoms and muscle tissue (using literature values). Solid lines indicate signal decay based on median relaxation parameters, and the shaded regions indicate possible relaxation across the range of reported T_1_ and T_2_ values. (E) Signal ratio between sodium phantoms and muscle tissue. Reducing the TE from 0.73 ms (full‐sinc acquisition) to 0.23 ms (half‐sinc acquisition, vertical lines) would be expected to result in a 2.5%–13.7% increase in calculated muscle aTSC. aTSC, apparent tissue sodium concentration.

Figure 2A shows ex vivo images for all three acquisition sequences. aTSC in porcine muscle was calculated as 25 ± 1 mM from 3D and 2D half‐sinc data. However, 2D full‐sinc data resulted in lower aTSC values of 24 ± 1 mM (Figure 2B). When relaxation correction was performed, minimal changes were observed in aTSC for 3D (+0.1 mM) and half‐sinc data (+0.2 mM). However, the full‐sinc signal correction resulted in an increase of 2.0 mM in aTSC (Figure 2B), resulting in higher aTSC than 3D and half‐sinc data.

**FIGURE 3:**
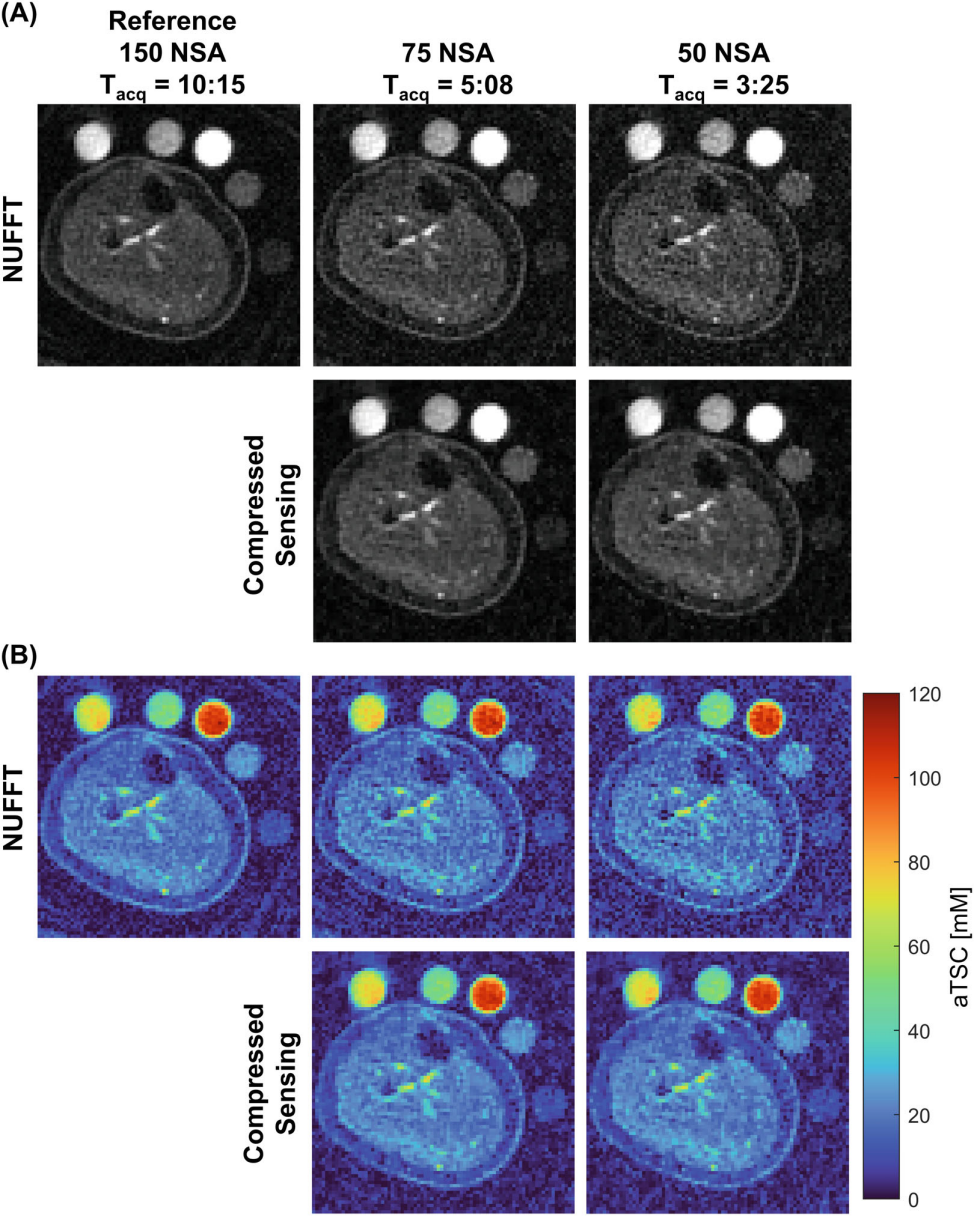
(A) Example in vivo NUFFT reference image (150 NSAs) and images retrospectively reconstructed with 75 and 50 NSAs using NUFFT and CS (with optimal regularization weighting factor). (B) Corresponding aTSC maps. Equivalent T_acq_ (min:s) are provided for each NSA. CS, compressed sensing; NUFFT, nonuniform fast Fourier transform; NSA, number of signal averages; T_acq_, acquisition times.

**FIGURE 4:**
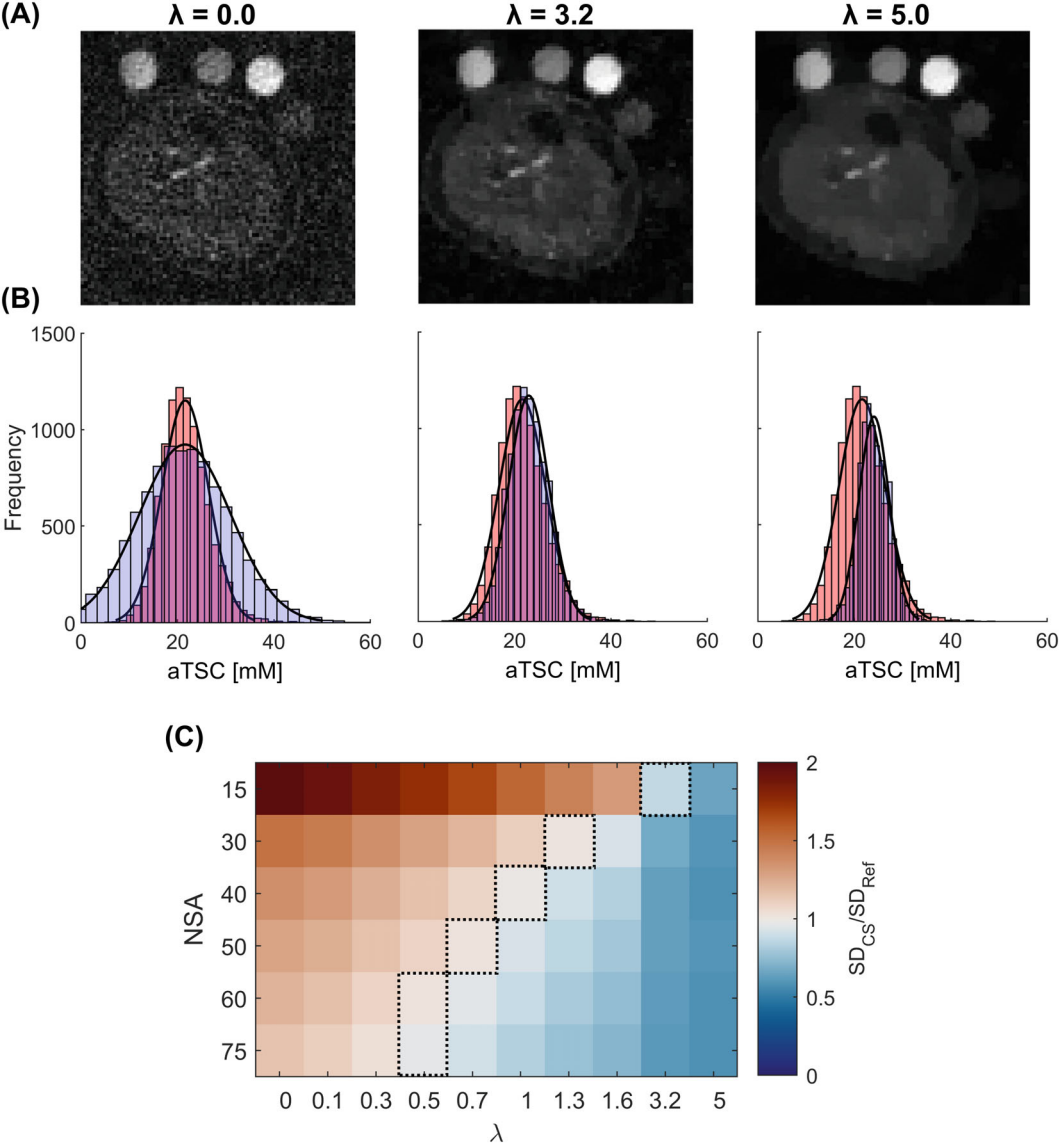
(A) Example in vivo images with 15 NSAs reconstructed with CS, showing the effect of different levels of λ. Unregularized reconstruction (λ = 0.0) produces images with extremely high levels of noise, which can be reduced with increasing λ (optimal λ = 3.2). However, the images become over‐smoothed with very high λ (λ = 5.0). (B) Histogram plots showing distribution of aTSC across pixels within the muscle ROI for images shown in A (blue) and the reference image (red, 150 NSAs, NUFFT). With low regularization, the distribution of pixels is broader than the reference image (SD_CS_/SD_Ref_ > 1) due to the high levels of noise. The distribution narrows as λ increases, and subsequently becomes narrower than the reference as the image becomes over‐smoothed (SD_CS_/SD_Ref_ < 1), while also introducing a bias in the mean aTSC. (C) Matrix showing ratio of SD of calculated aTSC in the muscle, between CS maps (SD_CS_), reconstructed with different NSAs and λ, and the reference map (SD_Ref_). Dashed lines indicate the optimal λ for a given NSAs (chosen where SD_CS_/SD_Ref_ ≈ 1). λ, regularization weighting parameter; Ref, reference; ROI, region of interest.

In silico modeling of sodium signals for the phantoms and muscle (Figure 2D) enabled the effect of different relaxation properties to be compared. At sequence‐specific TEs, the phantom/muscle signal ratio for the 3D, 2D full‐sinc, and half‐sinc acquisitions was 1.00, 0.93, and 0.99, respectively (Figure 2E). This translates to a median expected aTSC difference compared to the 3D data of 7.5% (range: 2.5%–24.0%) and 0.2% (range: 0.1%–0.3%) with the full‐sinc and half‐sinc, respectively, suggesting the bias observed in ex vivo full‐sinc data may be due to differences in TE.

#### 3.1.2 In vivo imaging

Similarly, in vivo aTSC was found to be significantly higher (*p* < 0.001) when quantified from half‐sinc compared to full‐sinc acquisitions (22 ± 2 and 19 ± 3 mM vs. 20 ± 2 and 17 ± 4 mM, for muscle and skin, respectively) (Figure 2C). This 10% difference is in agreement with the in silico model, suggesting the lower TE of half‐sinc excitation results in more accurate aTSC where relaxation correction is not feasible. Therefore, only half‐sinc data was carried forward for CS reconstruction (although it could be equally applied to full‐sinc data).

### 3.2 Optimization of compressed sensing on half‐sinc data

Reducing NSAs in in vivo half‐sinc data resulted in higher noise in NUFFT images (Figure 3A) (Supporting Information S4). Figure 4A,B (and Supporting Information S5) show the effect of CS with different regularization weighting on the distribution of aTSC within the muscle ROI. Although increasing λ reduces the width of the histogram of the muscle pixels (images become less noisy), very high λ results in excessive reduction in pixel variability, an over‐smoothed image, and a bias in muscle aTSC. The optimal λ values for different NSAs are presented in Figure 4C. Example images reconstructed with optimal λ across the range of NSAs and images reconstructed across the λ range are presented in Supporting Information S4 and S6, respectively.

CS reconstruction with optimal λ (CS‐λ) produced good image quality with as few as 50 NSAs (Figure 3) (Supporting Information S4). Therefore, images reconstructed with both 75 NSAs (halving of scan time compared to the 150 NSAs reference) and 50 NSAs were taken forward for muscle and skin aTSC quantification and image quality assessment.

### 3.3 aTSC quantification and image quality assessment

In vivo aTSC calculated within muscle and skin, as well as quantitative (estimated SNR and ES) and qualitative image quality metrics, are presented in Table 1. No significant differences in aTSC were observed between reference maps and maps with either 50 or 75 NSAs when reconstructed with the NUFFT or CS‐λ reconstruction (*p* > 0.9).

**TABLE 1:** Results comparing in vivo reference data (150 NSAs with NUFFT reconstruction) to the data retrospectively reconstructed with reduced NSAs (75 and 50), using NUFFT and CS‐λ. aTSC quantified in the muscle and skin of the calf, quantitative estimated SNR, quantitative edge sharpness, and qualitative image‐quality scores are shown from 2D half‐sinc data.

				75 NSAs					50 NSAs				
Metric			Reference (150 NSAs)	NUFFT	CS‐λ	*p* (Ref vs. NUFFT)	*p* (Ref vs. CS‐λ)	*p* (NUFFT vs. CS‐λ)	NUFFT	CS‐λ	*p* (Ref vs. NUFFT)	*p* (Ref vs. CS‐λ)	*p* (NUFFT vs. CS‐λ)
Accuracy	aTSC [mM]	Muscle	21.5 ± 1.9	21.7 ± 2.0	22.1 ± 2.0	1.0	0.99	1.0	21.7 ± 2.0	22.2 ± 2.0	1.0	0.98	1.0
		Skin	18.8 ± 3.2	18.8 ± 3.3	18.2 ± 3.4	1.0	1.0	1.0	19.0 ± 3.2	18.1 ± 3.3	1.0	1.0	0.99
Quantitative image scores	Estimated SNR	Muscle	7.0 ± 0.7	5.2 ± 0.5	7.5 ± 0.9	<0.001	0.64	<0.001	4.4 ± 0.4	6.5 ± 0.7	<0.001	0.50	<0.001
		Skin	6.1 ± 1.0	4.5 ± 0.7	6.1 ± 1.2	0.002	1.0	0.002	3.9 ± 0.5	5.3 ± 0.8	<0.001	0.26	0.008
	Edge sharpness [mM mm^−1^]		12.7 ± 0.5	12.7 ± 0.5	12.7 ± 0.5	1.0	1.0	1.0	12.8 ± 0.5	12.7 ± 0.4	1.0	1.0	1.0
Qualitative image scores	Ability to identify separate structures of the leg		3.2 ± 0.9	3.0 ± 0.7	3.8 ± 0.4	0.84	0.10	0.003	2.6 ± 0.5	3.5 ± 0.7	0.03	0.84	<0.001
	Perceptual noise		3.8 ± 0.4	3.1 ± 0.4	3.8 ± 0.4	<0.001	1.0	0.002	2.6 ± 0.5	3.5 ± 0.7	<0.001	0.26	<0.001

*Note*: Reference = NUFFT reconstructed half‐sinc data with 150 NSAs (scan time = 10:15 min:s). 75 NSAs scan time = 5:07 min:s, 50 NSAs scan time = 3:25 min:s. Data is presented as mean ± SD.

Abbreviations: λ, regularization weighting parameter; aTSC, apparent tissue sodium concentration; CS‐λ, compressed sensing with optimized λ; NSAs, number of signal averages; NUFFT, nonuniform fast Fourier transform.

Estimated SNR was similar in CS‐λ images and reference images (*p* > 0.2), and both were significantly higher than NUFFT images (*p* ≤ 0.008). No significant difference in ES was observed between CS‐λ, NUFFT, and reference aTSC maps (*p* = 1.0).

Example images with assigned scores from the qualitative scoring are presented in Supporting Information S7. CS‐λ images scored significantly higher than NUFFT images for perceptual noise (indicating less noise, *p* ≤ 0.002), but no difference was observed compared to reference images (*p* ≥ 0.2). Similarly, for the ability to identify separate structures of the leg, CS‐λ images scored significantly higher than NUFFT images (*p* ≤ 0.003), whereas no significant difference was measured with the reference (*p* ≥ 0.1).

## 4 DISCUSSION

In this study, we validated the use of half‐sinc excitation for 2D spiral UTE ^23^Na MRI reconstructed using CS. The main findings of this study were: (i) half‐sinc excitation led to improved accuracy in aTSC, ex vivo and in vivo; (ii) CS reconstruction improved qualitative and quantitative image quality of reduced NSAs data in vivo; and (iii) aTSC quantification was unaffected with reduced NSAs, irrespective of reconstruction (CS or NUFFT).

Ex vivo aTSC calculated without relaxation correction from 2D half‐sinc data was equivalent to that obtained from reference 3D UTE acquisitions, whereas full‐sinc data resulted in lower aTSC. This is expected due to the longer TE of the full‐sinc acquisition and minor differences in relaxation between calibration phantoms and tissue, and was supported by in silico modeling. Performing relaxation correction led to a minor increase in aTSC for half‐sinc and 3D data (≤0.2 mM). However, the full‐sinc correction was much larger (+2.0 mM), which may represent an overcorrection because the true tissue T_1_ and T_2_ are unknown. Unfortunately, T_1_ and T_2_ mapping is extremely time consuming and not feasible in a clinical setting.^33^, ^34^, ^35^ Consequently, the shorter TE of the half‐sinc acquisition, which minimizes T_2_ weighting and any resulting bias, is preferable to ensure greater aTSC quantification accuracy. For all techniques, good reproducibility was observed with a repeated measurement of one ex vivo sample (<0.4 mM difference in aTSC) (Figure 2B) and with calibration plots in all ex vivo samples (*N* = 4) (Supporting Information S1), with slightly better goodness of fit and reproducibility observed for half‐sinc data.

The main problem with half‐sinc acquisitions is the doubling of scan time; however, we have shown CS can be used to reconstruct images with fewer NSAs. Previous studies have investigated the use of CS in 3D ^23^Na MRI to remove undersampling artifacts, with early work showing underestimation of aTSC with CS reconstructions.^18^ More recent studies have demonstrated aTSC accuracy, with an undersampling factor up to 4 in the brain^17^ and leg.^20^ In this study, we found undersampling of data (with full NSAs) resulted in more residual artifacts than reduced NSAs (with full sampling; Supporting Information S8).

A vital aspect of CS is choosing the correct regularization weighting, with previous studies choosing based on visual inspection or structural similarity index (SSIM).^17^ In this study, we investigated methods based on pixel distribution in the aTSC maps, pixel distribution in the images, and SSIM (Supporting Information S9). Although the optimal regularization weighting was similar for all methods, matching pixel distribution in aTSC maps to the reference map was chosen because aTSC is the main clinical metric calculated from sodium images and SSIM has been shown to introduce contrast bias,^36^ which may result in errors in aTSC quantification. Increasing λ above the optimal level resulted in images becoming over‐smoothed, and a bias and loss of variability in aTSC. Compared to standard NUFFT images, CS did not change muscle or skin aTSC quantification. Nevertheless, estimated SNR was found to be improved compared to NUFFT images, whereas ES remained equivalent. These results demonstrate the denoising effect of CS without introducing image blurring, which is important when quantifying aTSC in small or thin structures such as the skin.

One of the main advantages of 2D imaging over 3D imaging is the ability to achieve higher in‐plane resolution and/or shorter scan times. All sequences used in this work have a higher spatial resolution (2.25 mm^2^) than typical sodium imaging (3–4 mm^2^),^37^, ^38^ but the half‐sinc 2D sequence has a significantly reduced acquisition time (≈10 min, with 150 NSAs) compared to the 3D sequence (24 mins). It should be noted that if images were acquired with a more conventional resolution (3–4 mm^2^), and equivalent SNR, the scan time for the 2D half‐sinc sequence could be reduced to ≈3 min (with 150 NSAs), and the 3D to ≈∼7.5 min.

### 4.1 Limitations

For the CS optimization, a 150 NSAs NUFFT 2D half‐sinc image was used as a reference. Although this image was visibly noisy, it provided the best estimate of in vivo aTSC values while reasonably limiting volunteer scan time. For clinical studies, true aTSC within tissue is not easily measured; however, in the future, preclinical studies may be conducted for full validation of aTSC accuracy.^39^

Accurate quantification of skin aTSC can be challenging due to its thin structure and partial volume effects caused by low spatial resolution. Despite increased spatial resolution compared to typical sodium imaging at 3 T,^37^, ^38^ skin aTSC was still lower than values reported at higher field strengths.^40^ It should also be noted that drawing ROIs directly onto sodium images may introduce bias; it may be more accurate to draw ROIs on registered high‐resolution proton images. Exact registration, however, can be challenging.

Another limitation of this method is the thick slices used. This is reasonable in the calf, where anatomical structure changes are small along the slice axis, and enables increased SNR and consequently higher in‐plane resolution. However, it may not be suitable for other applications. In this case, slice thickness can be reduced, and in‐plane resolution decreased to maintain SNR. Furthermore, this study only acquired a single 2D slice through the calf. For imaging other anatomy, such as kidney or brain, full coverage of the organ may be required, thus necessitating the use of 2D multi‐slice or 3D methods.

## 5 CONCLUSION

2D ^23^Na MRI using half‐sinc excitation and CS enables muscle and skin aTSC quantification in vivo in the calf within a reasonable scan time. Reducing scan time to ≤5 min will improve patient comfort and compliance and enables inclusion of ^23^Na MRI as part of a comprehensive quantitative MRI protocol while keeping the overall scan time short. By improving image quality and reducing the likelihood of motion artifacts while maintaining high resolution, this technique has the potential to improve diagnostic accuracy and monitoring of patients with diseases affecting sodium homeostasis.

## FUNDING INFORMATION

r.r.b. is funded by the University College London Hospitals (UCLH) Biomedical Research Centre (BRC) (grant BRC870/CM/JS/10132). j.m.t. and j.a.s. are funded by the UK Research and Innovation (UKRI) Future Leaders Fellowship (grant MR/S032290/1). s.r. is supported by the Engineering and Physical Sciences Research Council (EPSRC)‐funded University College London (UCL) Centre for Doctoral Training in Intelligent, Integrated Imaging in Healthcare (i4health) (grant EP/S021930/1), and the Department of Health's National Institute for Health and Care Research (NIHR)‐funded BRC at UCLH. b.s.s. is supported by Wings for Life (grant 169111). c.g.w.k. receives funds from Horizon2020 (Research and Innovation Action Grants Human Brain Project [SGA3]) (grant 945539), the BRC (grant BRC704/CAP/CGW), the Medical Research Council (MRC) (grant MR/S026088/1), Ataxia UK and the Rosetrees Trust (grants PGL22/100041 and PGL21/10079). S.B.W. is supported by Kidney Research UK (grant RP_017_20190306).

## CONFLICT OF INTEREST STATEMENT

c.g.w.k. is a shareholder in Queen Square Analytics Ltd.

## Supporting information


**Supporting Information S1.** Goodness‐of‐fit of phantom linear calibrations performed in ex vivo experiments, from reference 3D and 2D full‐sinc and half‐sinc acquisitions.
**Supporting Information S2.** Example regions of interest used for aTSC quantification and quantitative image quality metrics.
**Supporting Information S3.** Composition and properties of sodium calibration phantoms.
**Supporting Information S4.** In‐vivo images and aTSC maps for a range of NSAs, with NUFFT and CS reconstruction.
**Supporting Information S5.** Histogram plots of in‐vivo aTSC in muscle, comparing CS to reference maps for the full range of NSAs and λ.
**Supporting Information S6.** Example aTSC maps reconstructed using CS with a range of λ values.
**Supporting Information S7.** Example in‐vivo images from qualitative scoring.
**Supporting Information S8.** Comparison of CS using undersampling or reducing NSAs.
**Supporting Information S9.** Different methods for optimizing the CS regularization weighting factor (λ).
