# Supplementary material for: 2D sodium MRI of the human calf using half‐sinc excitation pulses and compressed sensing
Source: Magn Reson Med. 2023 Oct 5;91(1):325–36. doi: 10.1002/mrm.29841 (PMC10962573; doi:10.1002/mrm.29841)
Supplement: Supplementary file 1 — Supporting Information S1. Goodness‐of‐fit of phantom linear calibrations performed in ex vivo experiments, from reference 3D and 2D full‐sinc and half‐sinc acquisitions. Supporting Information S2. Example regions of interest used for aTSC quantification and quantitative image quality metrics. Supporting Information S3. Composition and properties of sodium calibration phantoms. Supporting Information S4. In‐vivo images and aTSC maps for a range of NSAs, with NUFFT and CS reconstruction. Supporting Information S5. Histogram plots of in‐vivo aTSC in muscle, comparing CS to reference maps for the full range of NSAs and λ. Supporting Information S6. Example aTSC maps reconstructed using CS with a range of λ values. Supporting Information S7. Example in‐vivo images from qualitative scoring. Supporting Information S8. Comparison of CS using undersampling or reducing NSAs. Supporting Information S9. Different methods for optimizing the CS regularization weighting factor (λ). [file MRM-91-325-s001.docx]

SUPPORTING INFORMATION

**Supporting Information S1: Goodness-of-fit of phantom linear calibrations performed in ex-vivo experiments, from reference 3D and 2D full-sinc and half-sinc acquisitions**

The relationship between the nominal sodium concentration of the phantoms and the normalized ^23^Na MRI signal intensity from ex-vivo experiments (N=4) is shown in Figure S1. These sodium calibration plots were used to calculate apparent tissue sodium concentration (aTSC) maps from ^23^Na MRI images.

There is a good linear fit for 3D, 2D full-sinc, and 2D half-sinc methods, with R^2^>0.997, and good agreement between samples. Overall, the 2D half-sinc has a marginally higher R^2^ value and better agreement between experiments, suggesting the half-sinc acquisition may be most accurate and provide best reproducibility for aTSC quantification (with the assumption that the sample/tissue of interest has the same relaxation properties as the calibration phantoms).


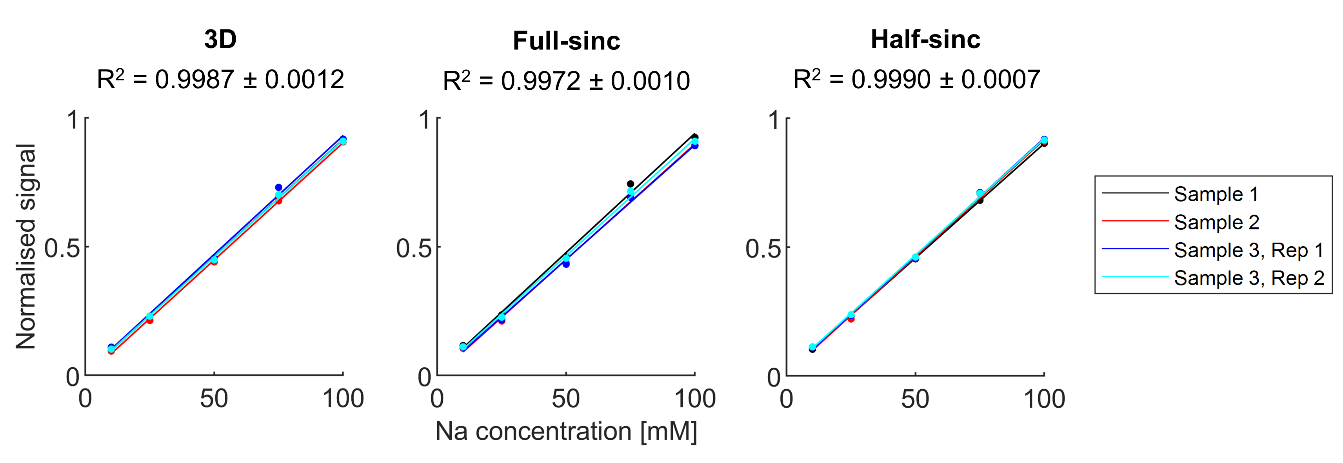


***Figure S1*** *Calibration plots from ex-vivo experiments and corresponding R^2^ values (mean±SD).*

**Supporting Information S2: Example regions of interest used for aTSC quantification and quantitative image quality metrics**

Figure S2A shows example regions of interest (ROIs) on ^23^Na MRI images from one subject. For aTSC quantification: red ROIs = calibration phantoms, blue ROI = skin, and purple ROI = muscle. For image quality metrics: yellow ROI = background area used for signal-to-noise ratio calculation, and green lines = four profiles (across the border of the 100mM phantom) used to calculate edge sharpness (ES).

Figure S2B shows an example line profile from one of the green lines (in Figure S2A) used to calculate ES. The left plot shows the aTSC value along the line (from outside to inside the phantom) in blue. The red line shows the Savitsky-Golay filtered profile (to remove the effect of noise), which is used to calculate the gradient (shown on the right). The ES value is taken as the maximum value from the gradient profile.


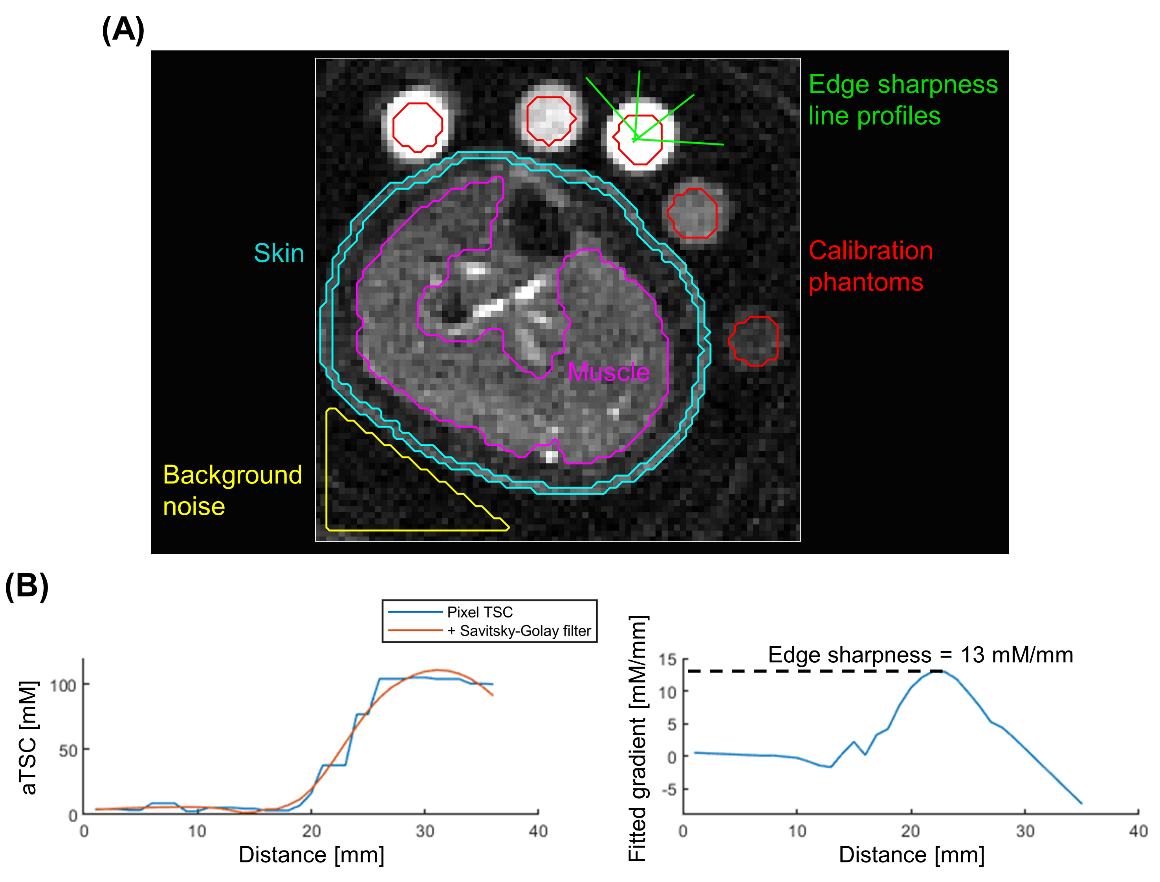


***Figure S2*** *(A) Example regions of interest for in-vivo muscle and skin apparent tissue sodium concentration (aTSC) quantification and image quality metrics. (B) Example line profiles for edge sharpness quantification: left plot shows aTSC value along the line, which is used to calculate the gradient, shown on the right.*

**Supporting Information S3: Composition and properties of sodium calibration phantoms**

The design of sodium calibration phantoms is extremely important for accurate quantification of tissue sodium concentration (TSC). When relaxation properties of phantoms and tissue do not exactly match, a bias may be introduced in the calculated TSC, as demonstrated in equation 2 in the main body of the paper. However, exactly matching all tissue relaxation parameters is not feasible, as different tissues have different relaxation properties (Figure S3), and even within one tissue type the relaxation properties may vary between healthy individuals and pathology.


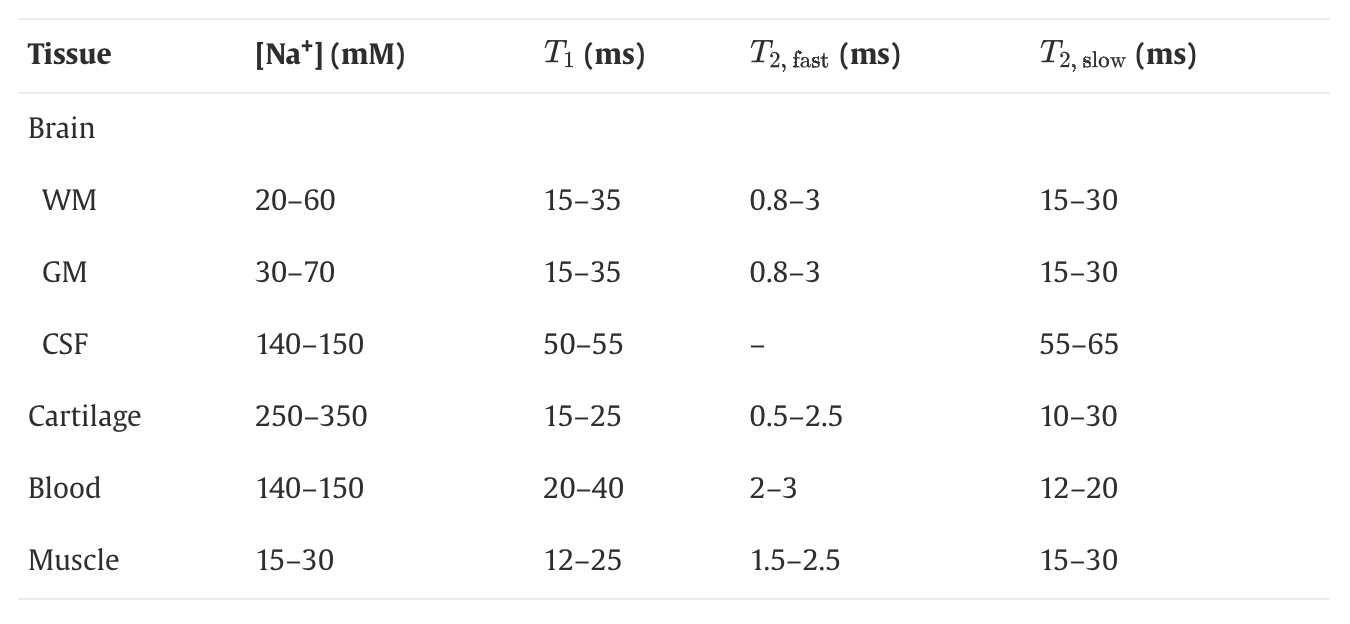


***Figure S3*** *Sodium concentrations and relaxation times in human tissue in-vivo, reproduced with permission from: Madelin G, Lee J, Regatte RR, Jerschow A. Sodium MRI: methods and applications. Progress in Nuclear Magnetic Resonance Spectroscopy 2014; 79:14–47.*

The calibration phantoms used in this work have been specifically designed for ^23^Na MRI as described by Rot et al.^1^ and are made from polyacrylamide gel (PAG) to ensure consistency across batches and stability over time. This is because traditional agarose gel phantoms are known to suffer from variability across batches and degradation.

The relaxation properties of these PAG phantoms have previously been measured by Rot, et al.^1^ using a non-localized free induction decay pulse-acquire spectroscopy protocol. For T_1_ measurements, a 180° inversion pulse was performed before acquiring the FID, with inversion times from 5–120 ms. The inversion recovery equation was fitted to the acquired data to give the mono-exponential T_1_ value. For T_2_ measurements, the FID was sampled directly, and a multi-exponential signal model was fitted to the data as described by Riemer et al.^2^ This model estimates the component fractions as well as the T_2_ relaxation times for the different components.

The resultant relaxation properties of the PAG phantoms were calculated to be: T_1_=27–32 ms, T_2S_=4–6 ms, T_2L_=17–24 ms, T_2S_:T_2L_ component ratio 0.7:0.3. Comparing these to muscle tissue, the measured T_2L_ values fall within the reported range for muscle, but the T_2S_ and T_1_ values are longer than for muscle tissue (with the T_1_ aligned with values in the brain, where sodium imaging is more commonly performed).

Despite differences in relaxation compared to muscle, the PAG phantoms are more closely matched than standard agar phantoms. Typical calibration phantoms made using 2–3% agar^3^ exhibit much longer relaxation times: T_1_=37–49 ms, T_2S_=6–10 ms, T_2L_=25–50 ms.^4–7^ Therefore, although not a perfect match, we consider the PAG phantoms to sufficiently mimic muscle tissue signal for use in this study.

**Supporting Information S4: In-vivo images and aTSC maps for a range of NSAs, with NUFFT and CS reconstruction**

Figure S4 shows example in-vivo data from one subject, demonstrating quality of images and aTSC maps for a range of signal averages (NSAs). The figure shows images reconstructed with a non-uniform fast Fourier transform (NUFFT), as well as compressed sensing (CS) using the optimal regularization weighting. Reducing the NSAs decreases the signal-to-noise ratio (SNR) in the NUFFT images, however CS is able to recover good quality images with NSAs≥50. With <50 NSAs image blurring and loss of information is observed.


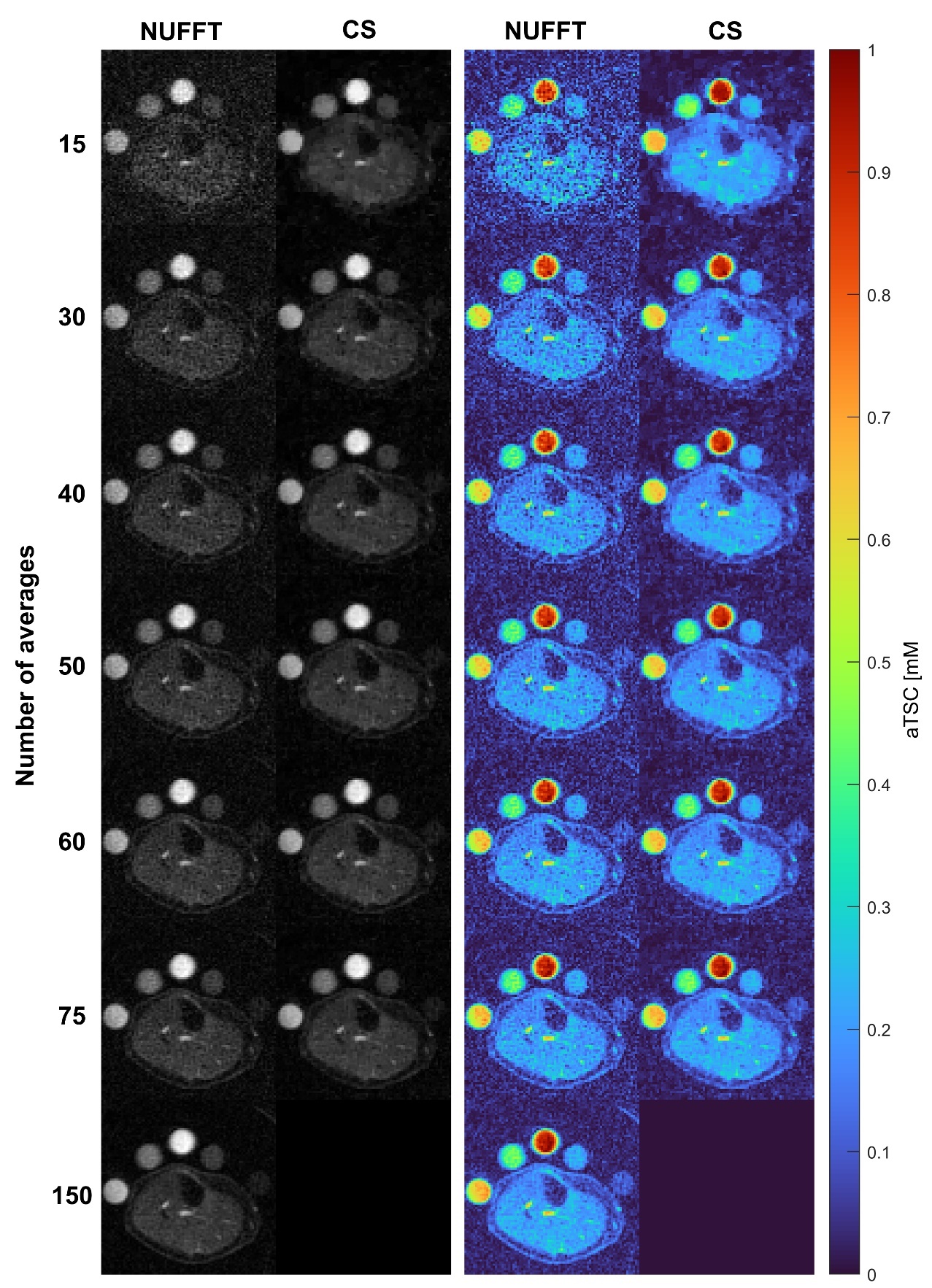


***Figure S4*** *In-vivo images and corresponding tissue sodium concentration maps for a range of signal averages, reconstructed with NUFFT and CS.*

**Supporting Information S5: Histogram plots of in-vivo aTSC in muscle, comparing CS to reference maps for the full range of NSAs and λ**

The optimal regularization weighting factor (λ) for a given NSAs was chosen by comparing the standard deviation (SD) of pixels in a muscle ROI in aTSC maps from the CS reconstruction (SD_CS_) with reference 150 NSAs NUFFT maps (SD_Ref_), using SD_CS_/SD_Ref_. This can also be understood by observing the histograms of in-vivo aTSC within the muscle ROI. Figure S5 shows the histograms for the reference maps (red) and CS reconstructed maps (blue) for the full range of NSAs and λ values tested.

It can be seen that where λ=0, with fewer NSAs the aTSC distribution across the pixels becomes broader compared to the reference, as the images exhibit more noise. As NSAs increase, the distribution narrows as the maps become less noisy.

Similarly, it can be seen that for a given NSA, as λ increases the variability in aTSC reduces and the images become smoothed. Where λ is too high (over-regularization), aTSC variability becomes less than that of the reference and the maps become overly smoothed, which also results in a shift/bias in mean aTSC.


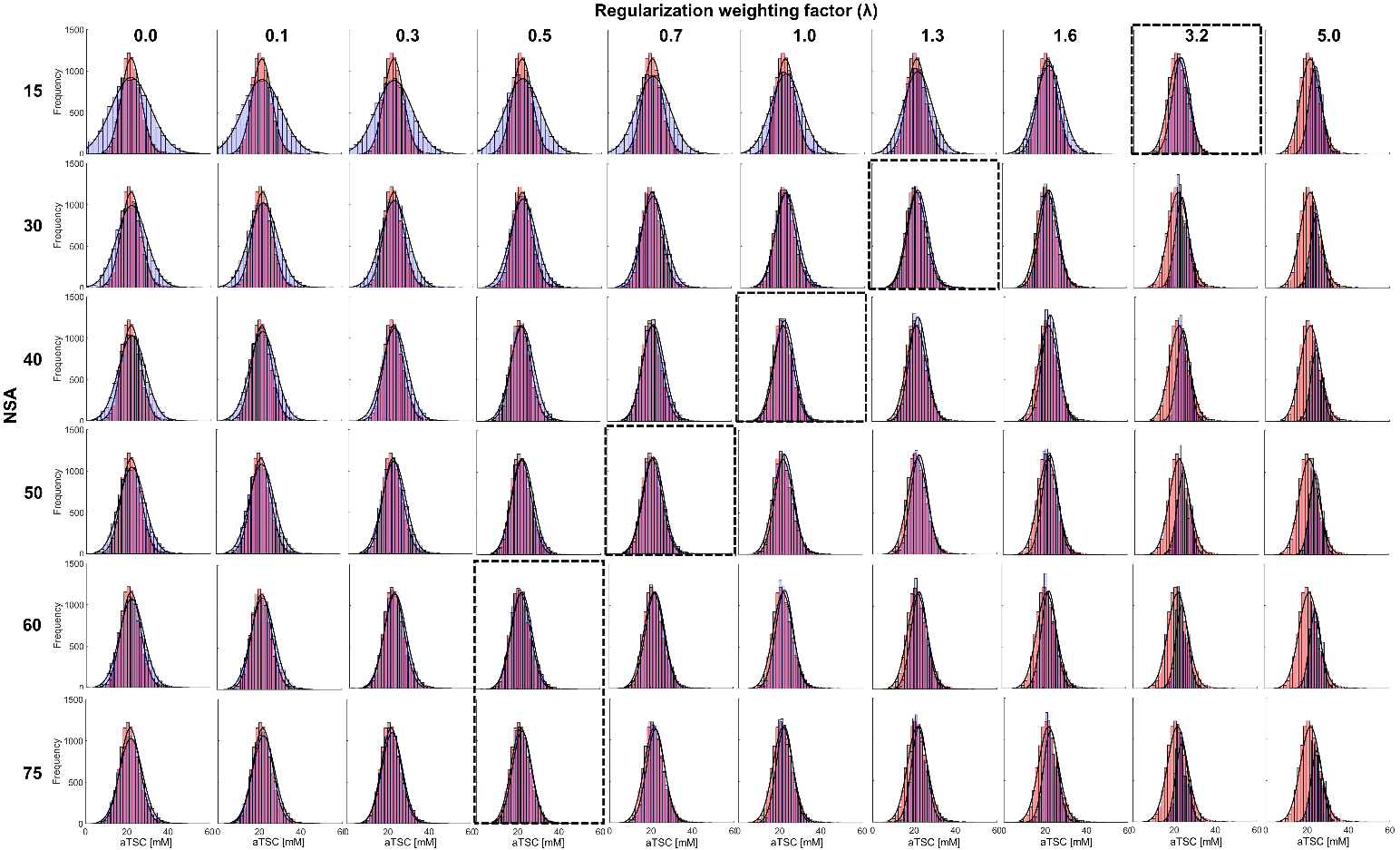
In this study, the optimum λ was taken where the aTSC distribution was most similar to the reference (SD_CS_/SD_Ref_ ~ 1); these are highlighted by the dashed lines in Figure S5. A summary of these data is provided in Figure 4 in the main body of the paper.

***Figure S5*** *Histograms of in-vivo tissue sodium concentration calculated within the muscle of the reference maps (red) and compressed sensing reconstructed maps (blue) for the full range of signal averages (NSAs) and λ values tested.*

**Supporting Information S6: Example aTSC maps reconstructed using CS with a range of λ values**

As discussed in Supporting Information S5, when using low λ values for CS, images and aTSC maps remain noisy, whereas with increasing λ images become less noisy, before becoming oversmoothed. Figure S6 shows example in-vivo aTSC maps from one subject, with 75 NSAs reconstructed using CS with different λ values. For 75 NSAs, optimal λ=0.5 and images become oversmoothed when λ>1.3.


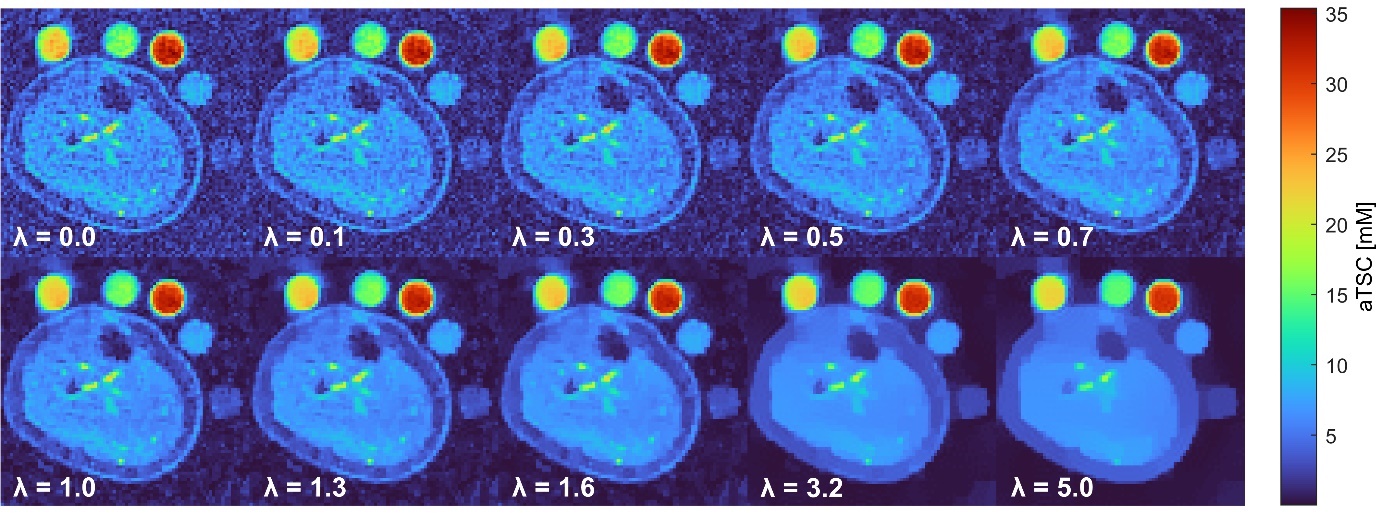


***Figure S6*** *Example in-vivo apparent tissue sodium concentration (aTSC) maps reconstructed with 75 signal averages (NSAs) and total variation-regularized compressed sensing across a range of regularization weighting factors (λ). The optimum λ for 75 NSAs was 0.5.*

**Supporting Information S7: Example in-vivo images from qualitative scoring**

Figure S7 shows example NUFFT and CS images (with optimal λ) which were qualitatively scored between 2 and 4 (the range in which most images were scored). Image quality was assessed in two categories: perceptive noise (upper panel) and ability to identify separate structures of the leg (lower panel). Images were scored by two independent observers. * Indicates images which were assigned the presented score by just one observer; otherwise, images were scored the same by both observers.

**
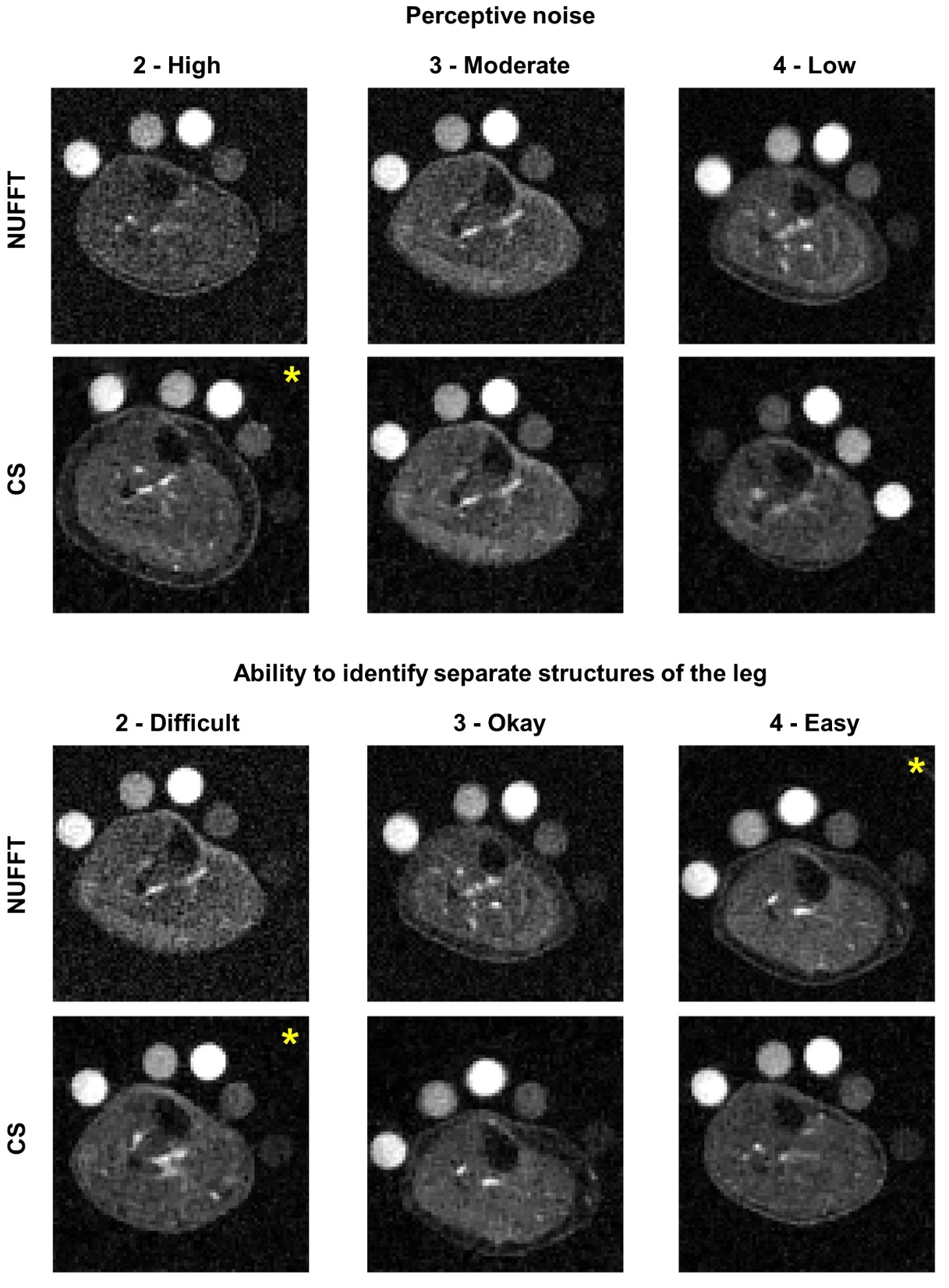
**

***Figure S7*** *Example in-vivo images scored between 2 and 4 for the different categories from the qualitative scoring.*

**Supporting Information S8: Comparison of CS using undersampling or reducing NSAs**

When halving the scan time of the half-sinc acquisition, data may be undersampled (alternate spiral interleaves, all NSAs) or NSAs reduced (all spiral interleaves, half NSAs). The effects of these different approaches are presented in Figure S8, for both NUFFT and CS reconstructions. When undersampling ×2, residual artefacts are present in the NUFFT image (Figure S8A), and although CS significantly removes the artefacts, the image quality is poor due to residual blurring (Figure S8B). Alternatively, images reconstructed with half the number of NSAs show no artefacts with the NUFFT reconstruction (Figure S8C, although lower SNR than the full acquisition) or CS reconstruction (Figure S8.1D).


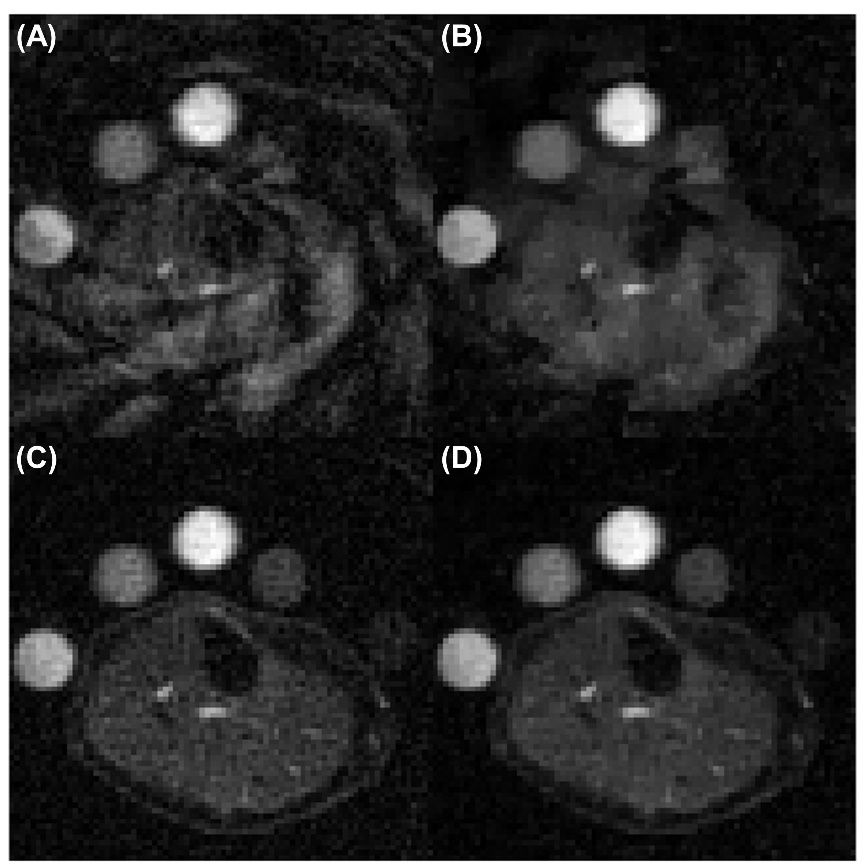


***Figure S8*** *Undersampled k-space (×2) with NSAs=150, reconstructed using NUFFT (A) and CS (B). Fully sampled k-space with half NSAs (75), reconstructed with NUFFT (C) and CS (D).*

**Supporting Information S9: Different methods for optimizing the CS regularization weighting factor (λ)**

Different approaches were investigated for choosing the optimal λ for CS reconstruction of the in-vivo 2D half-sinc data These included:

1. Comparing the standard deviation (SD) of pixels in a muscle ROI in aTSC maps from the CS reconstruction (SD_CS_) with reference 150 NSAs NUFFT maps (SD_Ref_), using SD_CS_/SD_Ref_ (as described in Supporting Information S5). The optimal value was chosen where SD_CS_/SD_Ref_ ~1.
2. Comparing the standard deviation of pixels in a muscle ROI in images from the CS reconstruction (SD_image_CS_) with reference 150 NSAs NUFFT images (SD_image_Ref_), using SD_image_CS_/SD_image_Ref._ The optimal value was chosen where SD_image_CS_/SD_image_Ref_ ~1.
3. Calculating SSIM between the CS and reference images. The optimal value was chosen where SSIM was highest.

Figure S9 shows these three metrics, calculated for data reconstructed for a range of NSAs and λ values. The results are presented as matrices, with dashed lines indicating the optimal λ for a given NSAs.

It can be seen that similar optimal λ results are obtained with all three metrics, however in this study, λ values were taken from matching pixel distribution in aTSC maps to the reference map (method 1 above), as aTSC is the main clinical metric calculated from sodium images and SSIM has been shown to introduce contrast bias which could affect aTSC quantification.^8^


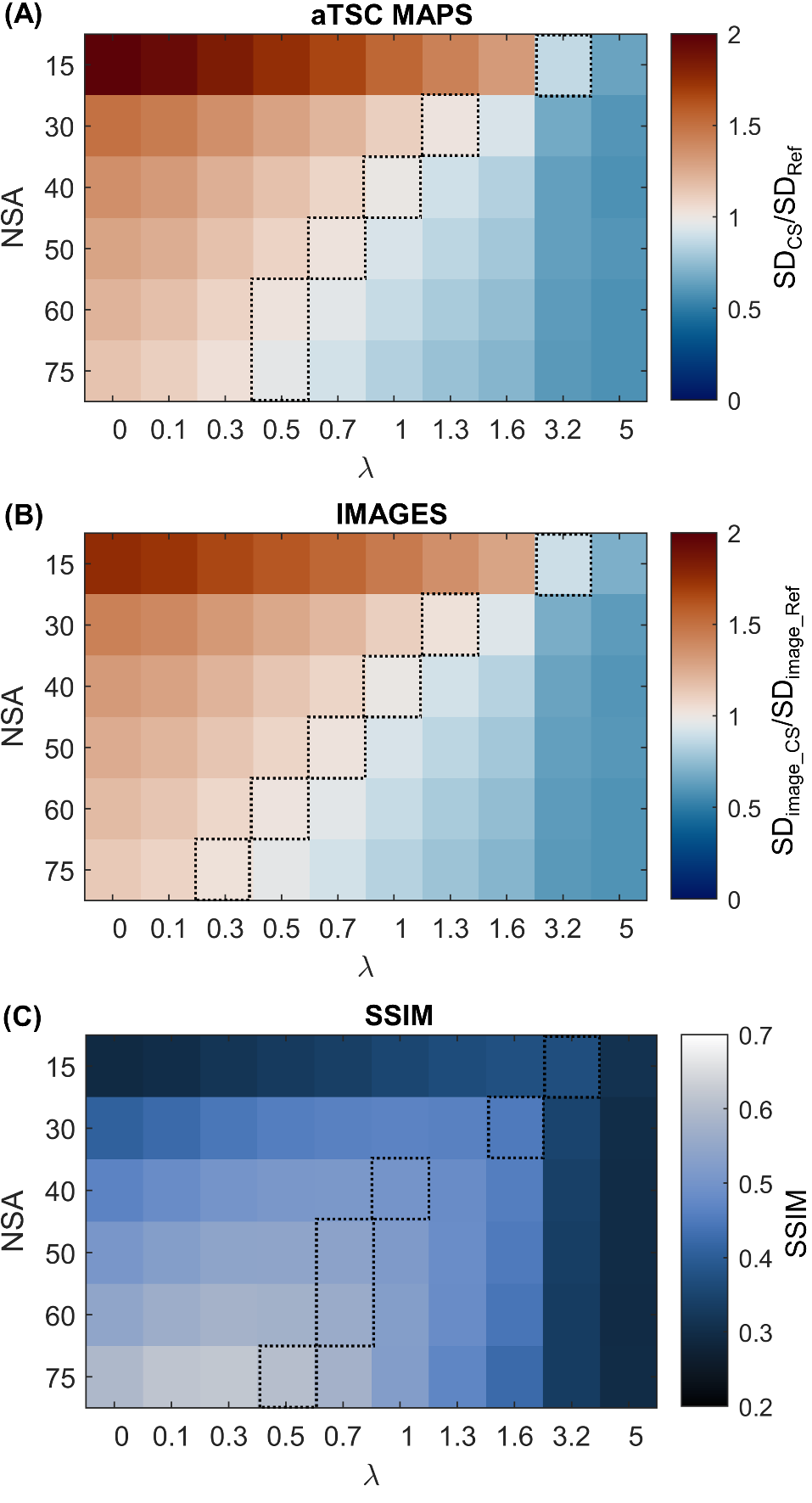


***Figure S9*** *(A) Matrix showing ratio SD_CS_/SD_Ref_ as calculated from SD of muscle pixels in aTSC maps. (B) Matrix showing ratio SD_image_CS_/SD_image_Ref_ as calculated from SD of muscle pixels in images. (C) Matrix showing structural similarity index (SSIM) calculated between the reference image and CS images. Dashed lines indicate the optimal λ for a given NSAs.*

**References**

1. Rot S, Oliver-Taylor A, Golay X, Solanky B, Gandini Wheeler-Kingshott CAM. Towards standardising quantification in 23Na-MRI by synthetic polyacrylamide gel phantoms. In: *Proceedings of the 31st Annual Meeting of ISMRM*. London, UK; 2022:1281.

2. Riemer F, Solanky BS, Wheeler-Kingshott CAM, Golay X. Bi-exponential 23Na T2* component analysis in the human brain. *NMR Biomed*. 2018;31(5):e3899.

3. Madelin G, Lee J, Regatte RR, Jerschow A. Sodium MRI: methods and applications. *Prog Nucl Magn Reson Spectrosc*. 2014;79:14-47.

4. Kratzer FJ, Flassbeck S, Nagel AM, et al. Sodium relaxometry using 23Na MR fingerprinting: a proof of concept. *Magn Reson Med*. 2020;84(5):2577-2591.

5. Kratzer FJ, Flassbeck S, Schmitter S, et al. 3D sodium (23Na) magnetic resonance fingerprinting for time-efficient relaxometric mapping. *Magn Reson Med*. 2021;86(5):2412-2425.

6. Coste A, Boumezbeur F, Vignaud A, et al. Tissue sodium concentration and sodium T1 mapping of the human brain at 3 T using a Variable Flip Angle method. *Magn Reson Imaging*. 2019;58:116-124.

7. Blunck Y, Josan S, Taqdees SW, et al. 3D-multi-echo radial imaging of 23Na (3D-MERINA) for time-efficient multi-parameter tissue compartment mapping. *Magn Reson Med*. 2018;79(4):1950-1961.

8. Kotevski Z, Mitrevski P. Experimental Comparison of PSNR and SSIM Metrics for Video Quality Estimation. In: Davcev D, Gómez J, eds. *ICT Innovations 2009*. Berlin, Heidelberg: Springer; 2010:357-366.
